# The Lateralization of Speech-Brain Coupling Is Differentially Modulated by Intrinsic Auditory and Top-Down Mechanisms

**DOI:** 10.3389/fnint.2019.00028

**Published:** 2019-07-17

**Authors:** M. F. Assaneo, J. M. Rimmele, J. Orpella, P. Ripollés, R. de Diego-Balaguer, D. Poeppel

**Affiliations:** ^1^Department of Psychology, New York University, New York, NY, United States; ^2^Department of Neuroscience, Max Planck Institute for Empirical Aesthetics, Frankfurt, Germany; ^3^Departament de Cognició, Desenvolupament i Psicologia de l’Educació, University of Barcelona, Barcelona, Spain; ^4^Catalan Institute for Research and Advance Studies, Barcelona, Spain; ^5^Cognition and Brain Plasticity Unit, IDIBELL, L’Hospitalet de Llobregat, Spain; ^6^Institute of Neuroscience, University of Barcelona, Barcelona, Spain

**Keywords:** asymmetrical sampling, brain to stimulus synchronization, MEG (magnetoencephalography), speech perception, speech envelope tracking

## Abstract

The lateralization of neuronal processing underpinning hearing, speech, language, and music is widely studied, vigorously debated, and still not understood in a satisfactory manner. One set of hypotheses focuses on the temporal structure of perceptual experience and links auditory cortex asymmetries to underlying differences in neural populations with differential temporal sensitivity (e.g., ideas advanced by Zatorre et al. (2002) and Poeppel (2003). The Asymmetric Sampling in Time theory (AST) (Poeppel, 2003), builds on cytoarchitectonic differences between auditory cortices and predicts that modulation frequencies within the range of, roughly, the syllable rate, are more accurately tracked by the right hemisphere. To date, this conjecture is reasonably well supported, since – while there is some heterogeneity in the reported findings – the predicted asymmetrical entrainment has been observed in various experimental protocols. Here, we show that under specific processing demands, the rightward dominance disappears. We propose an enriched and modified version of the asymmetric sampling hypothesis in the context of speech. Recent work (Rimmele et al., 2018b) proposes two different mechanisms to underlie the auditory tracking of the speech envelope: one derived from the intrinsic oscillatory properties of auditory regions; the other induced by top-down signals coming from other non-auditory regions of the brain. We propose that under non-speech listening conditions, the intrinsic auditory mechanism dominates and thus, in line with AST, entrainment is rightward lateralized, as is widely observed. However, (i) depending on individual brain structural/functional differences, and/or (ii) in the context of specific *speech listening* conditions, the relative weight of the top-down mechanism can increase. In this scenario, the typically observed auditory sampling asymmetry (and its rightward dominance) diminishes or vanishes.

## Introduction

Considerable advances in our understanding of the neural basis of speech processing have been made in the last decade. There is emerging consensus about a functional dissociation of the neuronal substrate underlying speech processing into ventral and dorsal pathways (Hickok and Poeppel, 2007; Saur et al., 2008; Rauschecker and Scott, 2009; Friederici, 2012) increasing evidence suggests an important role of both hemispheres (albeit contributions differ depending on the processing levels) (Binder et al., 2000; Cogan et al., 2014; Sammler et al., 2015) and the crucial role played by the sensorimotor circuitry during verbal learning and speech comprehension processes (Lopez-Barroso et al., 2011, 2013) is well-established. There are, to be sure, open questions and unsolved puzzles. Here we address controversial findings regarding hemispheric lateralization in the auditory cortex during the processing of speech. We propose that the differential contribution of both hemispheres to the processing of the speech acoustics reflects intrinsic attributes of the neural populations in the auditory cortex as well as modulation by top-down influence from non-auditory cortical areas. We provide new neurophysiological data supporting these claims.

Based on early foundational work (Giraud et al., 2000; Ahissar et al., 2001; Rimmele et al., 2018a) followed by a number of recent experiments (Luo and Poeppel, 2007; Kubanek et al., 2013; Ding et al., 2014; Crosse et al., 2015; Rimmele et al., 2015), it is now established that during speech comprehension low-frequency neural activity is *entrained* by connected speech, and in particular by attributes of the speech envelope. Neuronal entrainment (or speech tracking) denotes the alignment of the neuronal excitability phase of slow oscillations in auditory cortex with slow energy fluctuations in the speech acoustics. Crucially, entrainment to speech has been argued to have causal force (Doelling et al., 2014; Ghitza, 2014) (rather than being epiphenomenal), and, accordingly, the associated neurophysiological mechanisms have received much attention. However, there are controversial findings in this growing literature that challenge existing explanations.

One hypothesized mechanism to account for the neuronal entrainment to speech and its hemispheric lateralization is the Asymmetric Sampling in Time model (AST) (Poeppel, 2003). AST postulates that there are two different temporal integration constants in non-primary auditory cortex that result from the *intrinsic* properties of local neuronal ensembles. An *asymmetric sampling in time* results from the right hemispheric auditory cortical structures having a larger population of neural ensembles with longer temporal integration windows [∼100 to 300 ms, i.e., roughly corresponding to the syllabic rate (Ding et al., 2017)] compared to the left. These temporal windows, or specifically their neural instantiation, is reflected in neuronal oscillatory activity (longer window: theta; shorter window: low gamma) that aligns with basic units of speech, viz. syllables (theta) and phonetic or segmental information (gamma). In accordance with this hypothesis, there exists a growing body of evidence supporting a rightward preference for the processing of more slowly modulated acoustic information (Boemio et al., 2005; Giraud et al., 2007; Abrams et al., 2008; Telkemeyer et al., 2009; Morillon et al., 2012) in this regard, the AST conjecture accords well with related hypotheses about hemispheric asymmetries in processing spectral versus temporal sound characteristics (Zatorre and Belin, 2001). As the proposed temporal integration constants relate closely to the intrinsic properties of the auditory cortex in each hemisphere (Zatorre et al., 2002; Poeppel, 2003), we refer to the neuronal oscillatory activity in auditory cortex as an *intrinsic mechanism*.

In spite of this evidence, a closer inspection of previous findings reveals that most of the studies that report a rightward lateralization of the processing of slow acoustic modulations rely on tasks with low language processing demands, typically using auditory stimuli such as non-speech signals (Zatorre and Belin, 2001; Boemio et al., 2005; Telkemeyer et al., 2009; Vanvooren et al., 2014) (e.g., modulated noise or pure-tone patterns), unattended speech (Abrams et al., 2008, 2009), streams of monosyllables (Doelling et al., 2014), or a small number of sentences repeated many times (Luo and Poeppel, 2007).

Furthermore, it has been shown that the strength of speech entrainment in the left (Ahissar et al., 2001; Zoefel et al., 2018), but not in the right (Peelle et al., 2013), auditory cortex covaries with speech intelligibility. Accordingly, another influence on auditory cortex entrainment to speech has been recently described. A set of experiments showed that top-down signals, coming from frontal areas, increase the synchronization between the auditory cortex and the stimulus envelope, particularly in the left hemisphere (Park et al., 2015, 2018; Morillon and Baillet, 2017). In light of these findings, Rimmele et al. (2018b) postulated that frontal areas modulate the intrinsic oscillatory activity of the auditory cortex on the basis of predictive cues in the speech signal, such as rate fluctuations, syntactic or semantic information, or motor production-related predictions, permitting a more flexible tracking of speech than that attained with oscillatory entrainment alone.

A natural question derives from this elegant proposal: how does the integration of these two mechanisms – i.e., *intrinsic auditory* and *externally driven* – modulate the canonical *rightward lateralization* of the slow frequency neuronal entrainment in auditory cortex? To answer this question, we built on the following observations: (i) most of the research that shows rightward lateralization relies on tasks with low language processing demands (Zatorre and Belin, 2001; Boemio et al., 2005; Abrams et al., 2008, 2009; Telkemeyer et al., 2009; Doelling et al., 2014; Vanvooren et al., 2014) (ii) the strength of speech entrainment in the left (Ahissar et al., 2001; Zoefel et al., 2018), but not in the right (Peelle et al., 2013) auditory cortex covaries *particularly* with speech intelligibility; (iii) frontal top-down signals can enhance the entrainment of the left auditory cortex to the speech envelope (Park et al., 2015, 2018; Federmeier, 2007); and (iv) a recent study demonstrates that speech tracking is affected by neurophysiological and neuroanatomical individual differences and that for a subset of the population – characterized by strong audio-motor interaction – the auditory tracking is balanced between hemispheres (i.e., the expected rightward asymmetry disappears) (Assaneo et al., 2019). Connecting these empirical observations, we propose that, while listening to non-speech stimuli, auditory entrainment principally reflects the *intrinsic auditory* mechanism, thus exposing the rightward hemispheric asymmetry. However, under specific speech-listening conditions, or due to neuronal functional and structural individual differences, the *externally driven* mechanism can affect the neuronal activity, mostly in the left hemisphere, equalizing the strength of entrainment across hemispheres. We apply new analyses to three published magnetoencephalography (MEG) datasets (Assaneo and Poeppel, 2018; Assaneo et al., 2019; Rimmele et al., 2019) to present new evidence to support these claims.

## Materials and Methods

The datasets used in this manuscript belong to three previously published experiments. Materials and methods of each experiment are briefly described below. For more detail see Assaneo and Poeppel (2018) for Experiment A, Assaneo et al. (2019) for Experiment B, and Rimmele et al. (2019) for Experiment C.

### Participants

All participants self-reported normal hearing and no neurological deficits, and all had normal structural MRI scans. Participants were paid for taking part in the different studies and provided written informed consent.

#### Experiments A and B

The protocol was approved by the local Institutional Review Board (New York University’s Committee on Activities Involving Human Subjects).

#### Experiment C

The protocol was approved by the local ethics committee of the University Hospital Frankfurt (Germany).

#### Experiment A

A cohort of 19 individuals participated in the study and two were removed – one was not able to perform the task, for the other one the MEG signal was too noisy. The analyzed sample consisted of 17 participants (9 males; mean age 28, range 20–40; 15 native speakers of American English and 2 native speakers of Spanish).

#### Experiment B

A group of 40 participants completed the experiment, the data from three was not analyze, since the acquired MEG signal was too noisy. The final database included 37 right handed participants (18 males; mean age, 30; age range, 21 to 55).

#### Experiment C

Twenty-one individuals participated in this study. Two participants were removed, because of outlier behavioral performance (accuracy < mean − 2 × SD) and because of technical issues (i.e., audio problems). The final sample comprised 19 right-handed German native speakers (*n* = 19) with no previous knowledge of Turkish (male: 9; mean age: 24.46 years; SD: 3.73 years).

### Task

#### Experiments A and B

In both experiments, participants passively listened to a set of syllable streams while their neural activity was recorded. At the end of each trial, participants indicated, by pressing a button, whether a given syllable had been presented. In Experiment A participants also completed a motor and an auditory localizer task.

#### Experiment C

During the MEG recording, participants were asked to listen attentively to sequences of di-syllabic German words (*Semantic Condition*) or Turkish pseudo-words (*Non-Semantic Condition*). Overall, 15 blocks were presented, each consisting of 210 trials (105 per condition). In total, each German and Turkish word (note that the syllables of the Turkish words were randomized) was repeated 15 times. Each block contained 29% trials with a target stimulus (i.e., a syllable repetition) equally distributed across conditions. After each trial, participants indicated the presence of a target stimulus with a button press.

### Stimuli

#### Experiment A

English syllables */ba/*, */wa/*, */ma/*, and */va/* were synthesized using an online text-to-speech software www.fromtexttospeech.com/. The stimulus intensity was normalized based on the amplitude root mean square and the signal was compressed to 120-ms duration using Praat software (Boersma, 2001). Trials contained 3 s of silence (baseline) followed by 6 s of syllables. Two syllables were randomly selected from out of the four syllables for each trial. The syllables were sequentially presented with an occurrence frequency of 0.7 for one and 0.3 for the other. Varying the syllable rate generated six different conditions of trials: 2.5, 3.5, 4.5, 5.5, and 6.5 syllables per second.

#### Experiment B

Five sets of syllable streams were generated using the MBROLA text-to-speech synthesizer (Bozkurt et al., 1996). All phonemes were equal in pitch (200 Hz), pitch rise and fall (with the maximum amplitude at 50% of the phoneme) and duration, which was set to 111 ms to get a presentation rate of 4.5 syllables per second. Each set lasted 2 min and consisted of 12 distinct syllables (unique consonant-vowel combinations).

#### Experiment C

In total, 134 German disyllabic words were selected from the CELEX lexical database (Baayen et al., 1995) and 134 Turkish disyllabic words from the TELL database^1^ [for details see Rimmele et al. (2019)]. German and Turkish syllables produced by a female German/Turkish bilingual speaker were recorded. The recordings were high-pass filtered at 60 Hz, compressed in duration (250 ms), and normalized in peak-amplitude and pitch contour (at 250 Hz). The two syllables of each word were concatenated to generate the German word stimuli. Di-syllabic pseudo-words were created by concatenating two syllables that were quasi-randomly selected from all Turkish syllable stimuli with equal probability of first/second syllable position. For each sequence, randomly selected disyllabic stimuli were concatenated (19 disyllabic stimuli per sequence). Overall, three different sets of sequences were created.

### Data Acquisition and Processing

#### Experiments A and B

Neural activity was recorded with a 157-channel whole-head axial gradiometer system (KIT, Kanazawa Institute of Technology, Japan) emplaced in a magnetically shielded room. The recordings were acquired at 1000 Hz. An online bandpass filter between 1 and 200 Hz and a notch filter at 60 Hz were applied.

In order to monitor the subject’s head position, five electromagnetic coils were attached and localized to the MEG sensors at the beginning of the experiment. The position of the coils with respect to three anatomical landmarks: nasion, and left and right tragus were determined using a 3D digitizer software (Source Signal Imaging, Inc.) and digitizing hardware (Polhemus, Inc.). This measurement was used to coregister the MEG data with the subjects’ anatomical magnetic resonance image (MRI).

Data processing and analyses were conducted using custom MATLAB code and the FieldTrip toolbox (Oostenveld et al., 2011). Noisy channels were visually rejected for each participant’s dataset. The continuous MEG recordings were submitted to two different procedures. First, a least squares projection was fitted to the data from the 2 min of *empty room* recorded at the end of each session. The corresponding component was removed from the recordings (Adachi et al., 2001). Second, the environmental magnetic field was measured with three reference sensors located away from the participant’s head, and was regressed out from the MEG signals using time-shifted PCA (de Cheveigné and Simon, 2007). The MEG signals were detrended and artifacts related to eyeblinks and heartbeats were removed using independent component analysis.

#### Experiment C

A 269-channel whole-head system (Omega 2000, CTF Systems Inc.) situated in a magnetically shielded room was used for the MEG recordings. A sampling rate of 1200 Hz, an online low pass filter (cut-off: 300 Hz), and online denoising (higher-order gradiometer balancing) were applied. The head position relative to the MEG sensors was continuously tracked and head displacement was corrected in the breaks using the fieldtrip toolbox (Stolk et al., 2013). The data were band-pass filtered off-line (1–160 Hz, Butterworth filter; filter order 4) and line-noise was removed using bandstop filters (49.5–50.5; filter order 4). Muscle, jump and slow artifacts were removed in a semi-automatic artifact detection procedure. Trials that contained head movements that exceeded a threshold (5 mm) were rejected. Sensors with high variance were rejected. Eye-blink, eye-movement and heartbeat-related artifacts were removed, using independent component analysis [infomax algorithm (Makeig et al., 1996)]. The data was first reduced to 64 components using principal component analysis. Trials with correct responses were selected and the trial number was matched between the conditions by randomly selecting trials of the condition with less trials (overall trial number, mean = 68.68, SD = 10.27).

### Structural MRI

#### Experiments A and B

High-resolution T1-weighted 3D volume MR data were acquired using a Siemens Allegra 3T and a Siemens Prisma 3T scanner for Experiment A and B, respectively. Each participant’s MRI data were preprocessed following the FieldTrip pipeline. Cortical reconstruction and volumetric segmentation were performed with the FreeSurfer image analysis suite.

#### Experiment C

Individual T1-weighted MRI scans were acquired (for all participants except for three). The standard Montreal Neurological Institute (MNI) template brain was used, in case an individual MRI was missing. MRIs were recorded on a 3 Tesla scanner (Siemens Magnetom Trio, Siemens, Erlangen, Germany) and anatomical landmarks (nasion, left and right pre-auricular points) were marked via Vitamin-E capsules. From the individual MRIs of all participants, probabilistic tissue maps (including cerebrospinal fluid white and gray matter) were retrieved using the FieldtTrip toolbox.

### Source Reconstruction

Different approaches were used to reconstruct the brain activity across experiments: cortically constrained MNE (Dale et al., 2000) in Experiment A, linearly constrained minimum variance beamforming (Nolte, 2003) in Experiment B, and Dynamic Imaging of Coherent Sources (Gross et al., 2001) (DICS) in Experiment C.

### Brain-to-Stimulus Synchronization

#### Experiments A and B

Synchronization was estimated by computing the phase locking value (PLV) between the brain activity and the cochlear envelope (Ding et al., 2017) of the perceived stream of syllables. Specifically, the PLV was computed using the following formula:$P\,L\,V=\frac{1}{T}\,\left|\sum_{t=1}^{T}e^{i\,\left(\theta_{1}\,\left(t\right)-\theta_{2}\,\left(t\right)\right)}\right|$, where *t* is the discretized time, *T* is the total number of time points, and *θ_1_* and *θ_2_* represent the phase of the brain activity and the cochlear envelope, respectively.

For Experiment A the PLV was computed within a frequency band of ±0.5 Hz around the syllable rate using windows of 2-s length and 1-s overlap. The percentage of change from baseline was estimated as the difference between the PLV computed for the stimulation window and the PLV computed for the baseline window divided by the latter. For Experiment B the PLV was computed within a frequency band from 3.5 to 5.5 Hz using windows of 1-s length and 0.5-s overlap. In both cases the results for all time windows were averaged separately for each condition obtaining one PLV per voxel and per subject.

Auditory entrainment was estimated by averaging the PLVs of all voxels within the auditory cortex. The method used to define this region of interest (ROI) varied along experiments. In Experiment A, it was functionally localized. In Experiment B, areas were anatomically defined as BA 41/41, TE 1.0 and TE 1.2 using the Brainnetome Atlas (Fan et al., 2016).

#### Experiment C

The speech envelope was computed for each acoustic trial by using the following procedure (Smith et al., 2002): the waveforms were filtered into 8 frequency bands, the Hilbert transform was applied for each band, and the absolute magnitude of the 8 analytic signals was averaged. The obtained speech envelope was downsampled to 500 Hz. The spectral complex coefficients at 4 Hz were computed trial-wise for the speech envelope and the MEG data with a 0.1111 Hz resolution, and coherence was computed between all sensors and the speech envelope. The data were projected to source space using a common filter (DICS; λ = 100%; 0.8 cm grid), and Fischer *z*-transformation was applied. Voxels of the left and right Heschl’s Gyrus were selected based on the *automated anatomical labeling* atlas (Tzourio-Mazoyer et al., 2002) (AAL).

### Connectivity Analysis

#### Experiment B

The connectivity between the left primary auditory cortex and the 34 regions within the left frontal lobe was estimated using the weighted phase lag index (wPLI). Regions were anatomically defined using the Brainnetome Atlas (Fan et al., 2016), and activity was averaged for all sources within the same region. Primary auditory cortex was defined as BA 41/41, TE 1.0 and TE 1.2. In accordance with the Brainnetome Atlas, the frontal lobe comprised 34 regions: medial BA 8, dorsolateral BA 8, lateral BA 9, medial BA 9, medial BA 6, dorsolateral BA 6, medial BA 10, dorsal BA 9/46, Inferior Frontal Junction, BA 46, ventral BA 9/46, ventrolateral BA8, ventrolateral BA 6, lateral BA 10, dorsal BA 44, Inferior Frontal Sulcus, caudal BA 45, rostral BA 45, opercular BA 44, ventral BA 44, medial BA 14, orbital BA 12/47, lateral BA 11, medial BA 11, BA 13, lateral 12/47, BA 4 head and face region, BA 4 upper limb region, caudal dorsolateral BA 6, BA 4 trunk region, BA 4 tongue and larynx region, caudal ventrolateral BA 6, BA 1/2/3 lower limb region, BA 4 lower limb region.

The wPLI was computed between the left primary auditory cortex activity and the signal originated in each region of the left frontal lobe. First, the cross-spectrum between signals was computed as *X*=*Z*_*iFrontalZ*_audLeft^*^, where *Z* represents the Morlet wavelet transform of the signal – centered at 4.5 Hz and with the number of cycles of the wavelet set at 9 (Lachaux et al., 2002). Next, the wPLI square estimator was computed as (Vinck et al., 2011):

$$Y=\frac{I\,m\,\left(X\right)}{\left|X\right|}$$

$$w\,P\,L\,I\,\left(f\right)=\frac{{\left|\sum_{t=1}^{T}Y\,\left(f,t\right)\right|}^{2}-\sum_{t=1}^{T}Y\,\left(f,t\right)}{{\left(\sum_{t=1}^{T}\left|Y\,\left(f,t\right)\right|\right)}^{2}-\sum_{t=1}^{T}Y\,{\left(f,t\right)}^{2}}$$

where *f* is the frequency, *t* is the discretized time, and *T* is the total number of time points.

#### Experiment C

Source space connectivity was computed by multiplying the spectral complex coefficients of each trial (single taper frequency transformation; 0.1111 Hz resolution) with a common filter (DICS; across 2 and 4 Hz), computed across conditions separately for each trial. The debiased weighted phase lag index (Vinck et al., 2011) (dwPLI) was computed between all voxels. Fischer *z*-transformation was applied to normalize the data prior to further analysis. The connectivity between the STG and IFG was computed by averaging the dwPLI values within each ROI. The ROIs were selected based on the AAL (Tzourio-Mazoyer et al., 2002) (*Temporal_Sup_L* and *Frontal_Inf_Tri_L*). The connectivity of the ROI with itself was set to zero.

### Data Exclusion Criteria

In all the analyses, data points exceeding two standard deviations were removed. In Experiment C only correct responses were analyzed.

## Results

### Experiment A: Rightward Dominance Disappears for Speech Rates Deviating From Most Natural During a Syllable Perception Task

**FIGURE 1:**
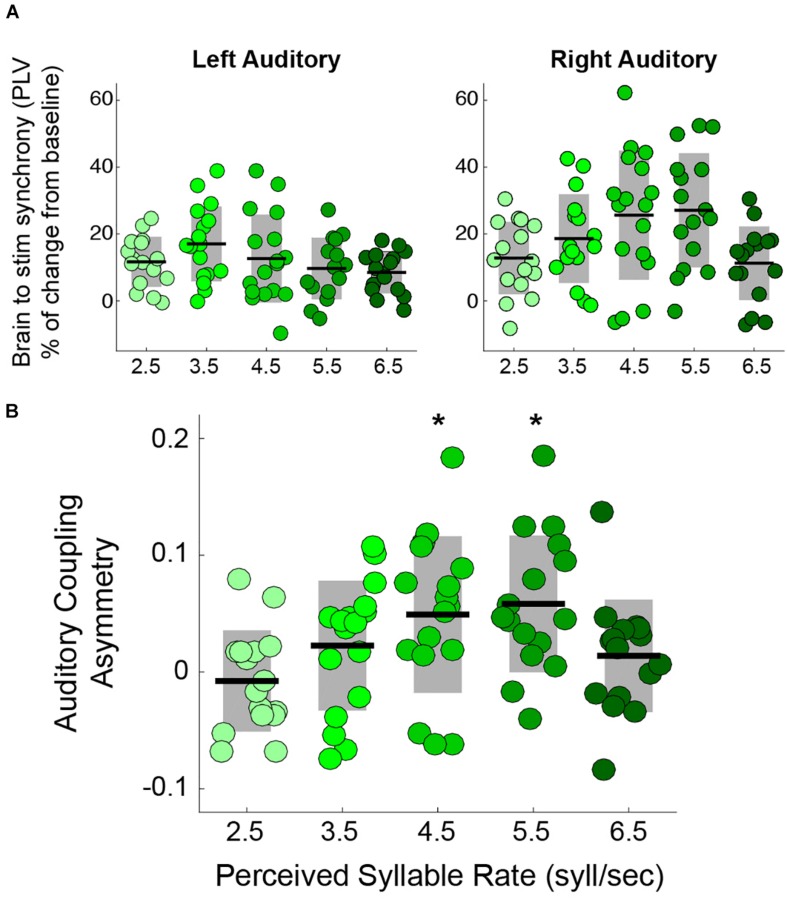
Rightward dominance is affected by speech rate during a syllable perception task. **(A)** PLV between auditory cortices and speech envelope, increment from resting state. Mean PLV around the syllable rate of each condition (syllable rate ± 0.5 Hz). Left auditory synchronization shows no change between conditions (Kruskal-Wallis test: χ^2^(4) = 5.6, two-sided *p* = 0.23). However, the right auditory cortex does (Kruskal-Wallis test: χ^2^(4) = 12.45, two-sided *p* = 0.014) (Adapted from Assaneo and Poeppel, 2018). **(B)** Auditory coupling asymmetry for the different syllable rate conditions: the degree of asymmetry is modulated by the syllable rate (Kruskal-Wallis test: χ^2^(4) = 13.63, two-sided *p* = 0.008). The asymmetry is significantly above zero only for 4.5 and 5.5 syllables per second. ^*^ Stands for two-sided *p* < 0.05 (Wilcoxon Signed-Rank test, FDR corrected). Dots: individual participants, the scattering in the *X*-axis is for visualization purposes. Black lines: mean across participants. Shaded region: SD. *N* = 17.

Previous studies showing the rightward dominance for speech envelope tracking focused on stimuli with a temporal modulation close to the natural syllable rate (Ding et al., 2017) (i.e., 4.5 syllables per second (Ding et al., 2017)). Here, we explored how the asymmetry is modified when the perceived syllable rate departs from the natural range by testing speech tracking at the typical rate and at the borders of the natural range. The auditory trials consisted of streams of syllables at different rates: 2.5, 3.5, 4.5, 5.5, and 6.5 syllables per second. We estimated, by means of the PLV, the synchronization between the activity in auditory cortex and the envelope of the perceived speech (see Materials and Methods). The results show that synchronization in the right auditory cortex, but not in the left, is modulated by the syllable rate (Figure 1A). Furthermore, we found that the auditory coupling asymmetry – defined as 2(*PLV*_*right*_-*PLV*_*left*_)/(*PLV*_*right*_+*PLV*_*left*_), positive values indicating a rightward asymmetry – is significantly different from zero only for the 4.5 and 5.5 syllables per second conditions (see Figure 1B).

### Experiment B: The Degree of Asymmetry Correlates With the Strength of Auditory-Frontal Connectivity During a Syllable Perception Task

**FIGURE 2:**
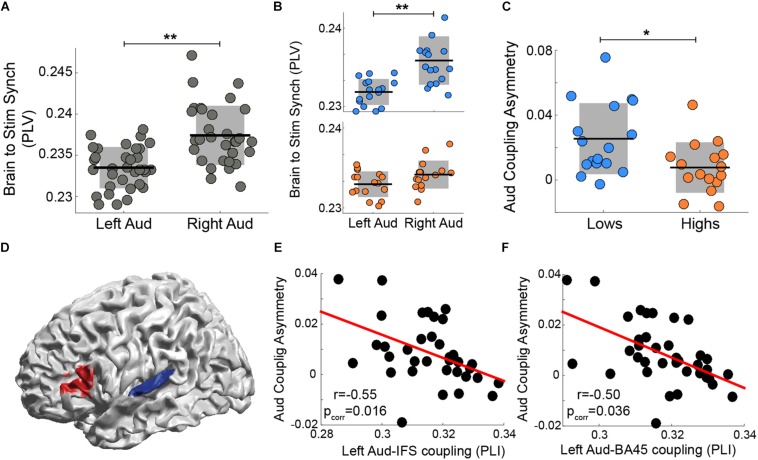
The degree of asymmetry correlates with the strength of auditory-frontal connectivity during a syllable perception task. Synchronization (PLV) between auditory cortex activity and the perceived speech envelope in left and right hemispheres: **(A)** all subjects pooled and **(B)** low synchronizers in the upper (blue) panel versus high synchronizers in the lower panel. **(C)** Auditory coupling asymmetry: comparison between groups. [**B,C** reanalyzed and replotted from Assaneo et al. (2019)] **(D)** Connectivity at 4.5 Hz (wPLI) between early auditory cortex (BA 41/41, TE 1.0 and TE 1.2 in blue) and frontal regions (caudal BA 45 and IFS in red) correlates with the degree of auditory-to-stimulus coupling asymmetry (Spearman correlation, two-sided *p* < 0.05, FDR-corrected). Scatter plots of the correlation between auditory-to-stimulus asymmetry and the wPLI between left auditory cortex and areas highlighted in red: **(E)** inferior frontal sulcus (IFS; *N* = 37) and **(F)** caudal BA 45 (*N* = 36). Orange/blue corresponds to high/low synchronizers, respectively. ^∗∗^ Stands for two-sided *p* < 0.005 (Wilcoxon signed-rank test), ^*^ for two-sided *p* < 0.05 (Mann-Whitney-Wilcoxon test). Dots: individual participants; in panels **A–C** the scattering in the *X*-axis is for visualization purposes. Black lines: mean across participants. Shaded region in panels **A–C**: SD. Red line: linear regression.

Assaneo et al. (2019) showed that, while participants listened to a stream of syllables, the rightward dominance of the envelope tracking is strongly diminished in a subset of the subject population. When the data from the whole participant cohort were pooled, the well-known asymmetry is evident (Figure 2A). However, when the cohort was segregated into two groups, subjects with high versus low performance on an audio-motor speech synchronization task, the envelope tracking is no longer significantly different across hemispheres for *high* performance participants (i.e., it is symmetrical; Figure 2B). Moreover, the asymmetry index was different between groups (see Figure 2C). We hypothesize that the asymmetry decreased due to stronger auditory-frontal interactions, that is, due to top-down influences from cortical regions external to the auditory cortex, especially to the left auditory cortex. Thus, by means of the wPLI analysis, we estimated the connectivity between the left auditory cortex and frontal regions at 4.5 Hz (see Materials and Methods). We found that left auditory to Brodmann area 45 (BA 45) and left auditory to inferior frontal sulcus (IFS) wPLI significantly correlated with the asymmetry index (Figures 2D–F). This result demonstrates that a stronger functional connectivity between left BA 45/IFS and left auditory cortex correlates with a more balanced (i.e., symmetrical) envelope tracking across hemispheres.

### Experiment C: Word-Level Linguistic Processing Reverses Rightward Dominance for the Envelope Tracking

**FIGURE 3:**
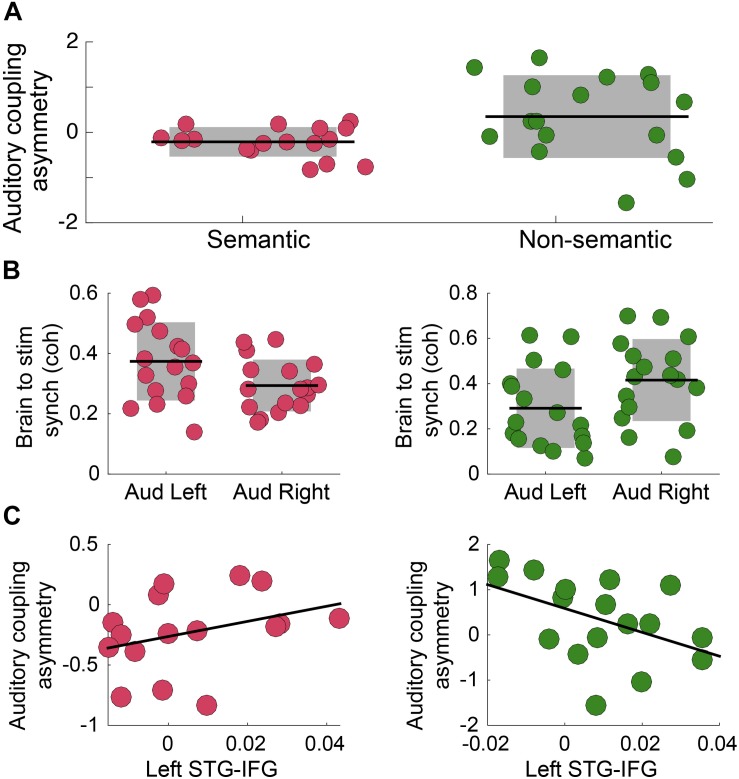
Semantic access reverses the classical rightward dominance for the envelope tracking. **(A)** Auditory coupling asymmetry index: comparison between conditions (*N* = 17; two-sided *p* = 0.035, paired Wilcoxon Signed-Rank test). **(B)** Coherence between *Heschl*’*s* gyrus (right and left hemispheres) activity and the auditory stimulus envelope, in a frequency band around 4 Hz. Left panel: *Semantic Condition* (*N* = 17; two-sided *p* = 0.010, paired Wilcoxon Signed Rank test). Right panel: *Non-Semantic Condition* (*N* = 17; two-sided *p* = 0.15, paired Wilcoxon Signed-Rank test). **(C)** Scatterplot of the auditory coupling asymmetry as a function of the connectivity between left STG- IFG, in a frequency band around 4 Hz. Left panel: *Semantic Condition* (*N* = 16; Spearman correlation coefficient *r* = 0.4, two-sided *p* = 0.12). Right panel: *Non-Semantic Condition* (*N* = 17; Spearman correlation coefficient *r* = –0.46, two-sided *p* = 0.063). In all panels: Pink/green correspond to *Semantic*/*Non-Semantic* (German words/Turkish pseudo-words) respectively, and dots: individual participants. In panels **A,B**: the shaded region represents SD, the black line the mean across participants and the dots scattering in the *X*-axis is for visualization purposes. In panel **C:** the black line represents the linear regression.

Previous research showed that speech intelligibility increases envelope tracking, particularly in the left auditory cortex (Peelle et al., 2013). Here we test how the asymmetry is modified by the presence of semantic information in the auditory stimulus. A cohort of German speakers performed an auditory syllable repetition detection task under two different conditions, defined by the type of stimuli: (i) *Non-Semantic*: Turkish disyllabic pseudo-word streams are presented (no lexical processing); and (ii) *Semantic*: German di-syllabic words streams are presented. This protocol allows us to explore how lexical-semantic processing modifies the asymmetry, independent of the task being performed – note that the task remained the same across conditions. First, we quantified the coherence between primary auditory cortex activity and the envelope of the perceived auditory stimulus, in a frequency band around the syllable rate (4 Hz). The results show that there was a significant difference in the asymmetry index for the two conditions of interest (Figure 3A). For the *Non-Semantic* condition, the asymmetry exposed a trend for the classical rightward dominance (right panel); for the *Semantic* condition the asymmetry was reversed (Figure 3B). Finally, we examined if there was a correlation between the auditory tracking and left fronto-temporal connectivity. For the *Non-Semantic* condition, the data showed the same trend as observed in Experiment B – the stronger the connectivity with the left auditory cortex, the weaker the rightward dominance (i.e., the more symmetrical the tracking was; see Figure 3C, right panel). However, the fronto-temporal connectivity did not seem to be directly related to the reversed asymmetry in the *Semantic* condition (see Figure 3C, left panel).

## Discussion

The neural architecture that forms the basis of speech processing is structurally and functionally complex, comprising a suite of computations that perform necessary subroutines on input and output processes. The tracking of the speech envelope by the auditory cortex has been proposed to be one of the basic mechanisms for the subsequent decoding of the signal. A common assumption is that envelope tracking is stronger in the right hemisphere than in the left – an effect that has been theoretically proposed (Poeppel, 2003) and experimentally demonstrated (Luo and Poeppel, 2007; Abrams et al., 2008; Doelling et al., 2014). However, here we show three examples in which the canonical rightward dominance is broken.

We hypothesize that in addition to the oscillatory activity intrinsic to the auditory cortex, speech tracking depends on input from other non-auditory brain regions, and we propose different functional roles for these mechanisms – i.e., *intrinsic auditory* and top down *externally driven* mechanisms. Lateralization depends on the extent to what these mechanisms are engaged to support a specific task.

### Right Hemisphere Tracking Preference Disappears for Non-natural Speech Rates During Syllable Perception

The AST theory (Poeppel, 2003) – as well as similar theories (Zatorre et al., 2002; Flinker et al., 2019) – builds on cytoarchitectonic differences between the (primary and non-primary) auditory cortices of the left and right hemispheres (Hutsler and Galuske, 2003). It proposes that those differences grant specific oscillatory properties to each hemisphere. Specifically, due to the biophysical properties of the neural populations, neuronal activity within the right auditory cortex shows characteristics of a neural oscillator with a natural frequency between 3 and 6 Hz. The basic features defining an oscillator are (Pikovsky et al., 2003): (i) it generates rhythmic activity at its own natural frequency, which is defined by the internal properties of the system; and (ii) it entrains to other oscillators or, as relevant in the speech case, synchronizes to external rhythmic stimuli, within a restricted range of frequencies close enough to its natural one.

Previous work found slow rhythmic neuronal activity during resting state within right auditory cortices (Giraud et al., 2007; Morillon et al., 2012), suggesting a neuronal population behaving as a low-frequency oscillator – consistent with criterion (i). Here, in line with criterion (ii), we found a tuning curve for the synchronization of the right auditory cortex (Figure 1A), with enhanced values for stimulus rates between 4 and 6 Hz. This was not found for the left auditory cortex, as presumably its natural frequency range differs. We propose that the brain-to-envelope coupling in the right hemisphere is driven by the oscillatory features of the auditory cortex, which are tuned to maximally resonate (in phase space) in response to frequencies close to the natural syllable rate (Ding et al., 2017). Thus, when the stimulation rate departs from the *natural* frequency of this area, the right cortex is less responsive and the tracking asymmetry disappears (Figure 1B). Furthermore, we hypothesize that the tuning curve obtained here is not inflexible; we believe that the range of entrainment could be extended under different task demands. According to previous proposals, the function of the right hemispheric speech tracking is related to the decoding of phonetic/spectral features of the audio signal (Zatorre and Belin, 2001; Poeppel, 2003). From a mathematical point of view, the entrainment range of an oscillator can be extended (Pikovsky et al., 2003) by increasing the strength of the coupling between the oscillator and the external driving force (in this case the auditory stimulus). Bringing those points together, we speculate that by modifying the goal of the task (e.g., a pitch perception or voice identity task, instead of the syllable perception task with a working memory component performed here) the right auditory-to-envelope coupling could be strengthened and the asymmetry could be recovered even for the less optimal modulation frequencies.

### During Syllable Perception Hemispheric Asymmetry Correlates With Auditory-Frontal Connectivity

Different functional roles have been attributed to speech envelope tracking: (i) segmentation of the input stream into temporal units of the appropriate granularity for subsequent decoding (Ghitza, 2014); (ii) extracting paralinguistic information; and (iii) integration of smaller phoneme-like units into larger syllable-like units for the subsequent phonological decoding. While (ii) is preferentially conducted by the right hemisphere, (iii) is more represented in the left (Giraud and Poeppel, 2012). We propose that the right hemisphere envelope tracking mostly reflects *intrinsic auditory* (bottom-up) oscillatory activity, while the left tracking (at the syllabic rate) is preferentially driven by cortical areas outside of auditory cortex (*externally driven*, top-down mechanism). Table 1 summarizes these conjectures.

**TABLE 1:** Different origins for the observed auditory-to-envelope synchronization.

	**Function**	**Dominant Hemisphere**	**Nature**
(i)	Paralinguistic/Phonetic	Right	Intrinsic auditory
(ii)	Phonological/Semantic	Left	Externally driven

*Speech envelope tracking is generated by distinct mechanisms. Each mechanism supports different functional roles and is asymmetrically represented across hemispheres. We define as *externally driven* a process relying on an interplay between the auditory cortex and other brain areas, while *intrinsic* refers to activity that reflects the internal oscillatory features of the auditory cortices.*

In Experiment B, on this view, the observed tracking reflects both *intrinsic auditory* and *externally driven* influences. On the one hand, since the temporal properties of the acoustic signal (i.e., the syllable rate of 4.5 Hz) match the natural frequency of the right auditory cortex, the right-lateralized intrinsic oscillatory mechanism is activated. On the other hand, the phonological processing required to complete the syllable perception task activates the *externally driven* mechanism. Thus, the envelope tracking lateralization is determined by the interplay between the recruited mechanisms. Our findings provide insight into how these influences interact and suggest that individual differences also play a role in the contribution of both mechanisms. Interestingly, while a part of the population shows the classic rightward dominance for the speech tracking (Figure 2A; note that when pooling together data from all participants this effect is observed), a subgroup of the population – with enhanced microstructural properties in the white matter pathways connecting the left auditory cortex with frontal regions (Assaneo et al., 2019)- displays no asymmetry in tracking (Figure 2B). We suggest that for this group, due to functional and structural differences, the influence of the *externally driven* mechanism is enhanced, equilibrating the tracking across hemispheres (Figure 2C). Moreover, the correlation between speech envelope tracking asymmetry and fronto-auditory connectivity (Figure 2D) supports the claim that *externally driven* top-down influences from the left frontal cortex to the left auditory one reverse the classical right hemispheric dominance. The same trend is found in Experiment C for the condition wherein a phonological task is performed on random streams of syllables (Turkish pseudo-words condition; note that this condition resembles the paradigm of Experiment B, Figure 3C right panel).

### Semantic Processing Reverses the Right Hemisphere Dominance

In Experiment C, as in the previously discussed study, speech tracking in both conditions presumably reflects both *intrinsic auditory* and *externally driven* contributions: on the one hand, the syllables are presented at a rate of 4 Hz – close to the natural frequency of the right auditory cortex – and on the other hand, the task requires phonological processing of the signal. During the condition in which German words were presented, additional lexical-semantic computations are necessarily performed (Rimmele et al., 2019).

Here, we show that, even though the task remains the same across conditions, and although the acoustic properties of the stimuli are similar, the asymmetry of the auditory tracking is reversed when semantic information is present (see Figures 3A,B left panel). We propose that semantic processing further enhances the envelope tracking performed by the left hemisphere, and thus reduces the right hemispheric dominance. This proposal aligns well with previous studies showing that the auditory to speech synchronization increases with intelligibility, specifically in the left hemisphere (Ahissar et al., 2001; Peelle et al., 2013; Rimmele et al., 2015).

Note that in Experiment C (in the German words condition), in spite of a reduced hemispheric asymmetry we found no correlation between fronto-temporal connectivity and the asymmetry index (as we do in Experiment B). Different reasons can underpin the dissimilarity between results. On one side, semantic access is a complex process – as compared to syllable perception – relying on large-scale brain networks (Hickok and Poeppel, 2000; Scott et al., 2002; Binder et al., 2009; Rodd et al., 2015) then, the asymmetry reduction can derive from the connectivity between temporal areas and a different region of the brain. On the other side, the task in Experiment B contains a working memory component, while the task in Experiment C does not load high on working memory. Further research is required to clarify the complex connectivity patterns between auditory cortex and other brain regions underpinning the hemispheric asymmetry and to investigate whether the correlation between the fronto-temporal connectivity and the asymmetry index might be related to working memory mechanisms.

It is worth noting that we employed distinct methods in the current work – different experimental designs, phase synchrony measurements and source reconstruction techniques. The results presented here derived from three already published studies. Thus, we chose to adopt for each analysis the approach applied in the original work. We believe that the fact that different experimental designs and methodological approaches show converging results further strengthens the reliability of our hypothesis.

To summarize, speech tracking (measured as auditory-cortex-to-speech envelope synchronization) is a complex process determined by an interplay between the intrinsic properties of the auditory cortices (Zatorre et al., 2002; Giraud et al., 2007) and top down influences from other non-auditory cortical areas related to different factors such as speech intelligibility, attention and/or acoustic properties of the perceived signal (Zion Golumbic et al., 2013; Ding et al., 2014; Zoefel and VanRullen, 2015; Bidelman and Howell, 2016). Moreover, individual differences in neural function and structure can also strongly affect the symmetry of the speech tracking between the hemispheres. Crucially, the *intrinsic auditory* and *externally driven* influences differently affect the hemispheric lateralization patterns of the speech tracking in the auditory cortex. Our findings illustrate the interaction between the different influences on speech tracking and suggest that the observed hemispheric lateralization patterns depend in subtle ways on task demands and the properties of the auditory signal. However, understanding the distinct origins of the assessed synchrony requires further research.

## Data Availability

The datasets for this manuscript are not publicly available because data available upon request. Requests to access the datasets should be directed to fassaneo@gmail.com.

## Author Contributions

All authors designed the research. MA, JR, JO, and PR acquired the data. MA and JR analyzed the data and wrote the manuscript.

## Conflict of Interest Statement

The authors declare that the research was conducted in the absence of any commercial or financial relationships that could be construed as a potential conflict of interest.

## Footnotes

1 http://linguistics.berkeley.edu/TELL/
