# Corrigendum: The Lateralization of Speech-Brain Coupling Is Differentially Modulated by Intrinsic Auditory and Top-Down Mechanisms

**DOI:** 10.3389/fnint.2020.00002

**Published:** 2020-02-04

**Authors:** M. F. Assaneo, J. M. Rimmele, J. Orpella, P. Ripollés, R. de Diego-Balaguer, D. Poeppel

**Affiliations:** ^1^Department of Psychology, New York University, New York, NY, United States; ^2^Department of Neuroscience, Max Planck Institute for Empirical Aesthetics, Frankfurt, Germany; ^3^Departament de Cognició, Desenvolupament i Psicologia de l'Educació, University of Barcelona, Barcelona, Spain; ^4^Catalan Institute for Research and Advance Studies, Barcelona, Spain; ^5^Cognition and Brain Plasticity Unit, IDIBELL, L'Hospitalet de Llobregat, Spain; ^6^Institute of Neuroscience, University of Barcelona, Barcelona, Spain

**Keywords:** asymmetrical sampling, brain to stimulus synchronization, MEG (magnetoencephalography), speech perception, speech envelope tracking

In the published article, there was an error regarding the affiliations for R. de Diego-Balaguer. As well as having affiliation 3, she should also have:

^4^ Catalan Institute for Research and Advance Studies, Barcelona, Spain
^5^ Cognition and Brain Plasticity Unit, IDIBELL, L'Hospitalet de Llobregat, Spain
^6^ Institute of Neuroscience, University of Barcelona, Barcelona, Spain

The authors apologize for this error and state that this does not change the scientific conclusions of the article in any way. The original article has been updated.

